# Effect of a Behavioural Intervention for Adoption and Maintenance of a Physically Active Lifestyle on Psychological Well-Being and Quality of Life in Patients with Type 2 Diabetes: The IDES_2 Randomized Clinical Trial

**DOI:** 10.1007/s40279-021-01556-0

**Published:** 2021-10-01

**Authors:** Antonio Nicolucci, Jonida Haxhi, Valeria D’Errico, Massimo Sacchetti, Giorgio Orlando, Patrizia Cardelli, Martina Vitale, Lucilla Bollanti, Francesco Conti, Silvano Zanuso, Giuseppe Lucisano, Stefano Balducci, Giuseppe Pugliese, for the Italian Diabetes and Exercise Study 2 (IDES_2) Investigators, Giuseppe Pugliese, Stefano Balducci, Massimo Sacchetti, Silvano Zanuso, Patrizia Cardelli, Antonio Nicolucci, Giuseppe Pugliese, Maria Cristina Ribaudo, Elena Alessi, Martina Vitale, Tiziana Cirrito, Lucilla Bollanti, Nicolina Di Biase, Filomena La Saracina, Stefano Balducci, Mario Ranuzzi Jonida Haxhi, Valeria D’Errico, Massimo Sacchetti, Giorgio Orlando, Luca Milo, Roberto Milo, Gianluca Balducci, Enza Spinelli

**Affiliations:** 1 Present Address: Centre for Outcomes Research and Clinical Epidemiology (CORESEARCH), Pescara, Italy; 2 Department of Clinical and Molecular Medicine, University of Rome La Sapienza, Via di Grottarossa, 1035-1039, 00189 Rome, Italy; 3 Diabetes Unit, Sant’Andrea University Hospital, Rome, Italy; 4 Metabolic Fitness Association, Monterotondo, Rome, Italy; 5 Present Address: Department of Human Movement and Sport Sciences, University of Rome ‘Foro Italico’, Rome, Italy; 6 Present Address: Department of Life Sciences, Research Centre for Musculoskeletal Science and Sports Medicine, Faculty of Science and Engineering, Manchester Metropolitan University, Manchester, UK; 7 Laboratory of Clinical Chemistry, Sant’Andrea University Hospital, Rome, Italy; 8 Present Address: Centre for Applied Biological and Exercise Sciences, Faculty of Health and Life Sciences, Coventry University, Coventry, UK; 9 Department of Clinical Pharmacology and Epidemiology, Consorzio Mario Negri Sud, S. Maria Imbaro, Italy; 10 Centre for Human Performance and Sport, University of Greenwich, Chatham Maritime, UK

## Abstract

**Background:**

Psychological well-being and quality of life (QoL) are important outcomes of lifestyle interventions, as a positive impact may favour long-term maintenance of behaviour change.

**Objective:**

This study investigated the effect of a behavioural intervention for adopting and maintaining an active lifestyle on psychological well-being and health-related QoL in individuals with type 2 diabetes.

**Methods:**

Three hundred physically inactive and sedentary patients were randomized 1:1 to receive 1 month’s theoretical and practical counselling once a year (intervention group, INT) or standard care (control group, CON) for 3 years. Psychological well-being and QoL, assessed using the World Health Organization (WHO)-5 and the 36-Item Short Form (SF-36) questionnaire, respectively, were pre-specified secondary endpoints. The primary endpoint was sustained behaviour change, as assessed by accelerometer-based measurement of physical activity (PA) and sedentary time.

**Results:**

WHO-5 and SF-36 physical and mental component summary (PCS and MCS) scores increased progressively in the INT group and decreased in the CON group, resulting in significant between-group differences (WHO-5: mean difference 7.35 (95% confidence interval (CI) 3.15–11.55), *P* = 0.0007; PCS 4.20 (95% CI 2.25–6.15), *P* < 0.0001; MCS 3.04 (95% CI 1.09–4.99), *P* = 0.0025). Percentage of participants with likely depression decreased in the INT group and increased in the CON group. PA volume changes were independently associated with WHO-5 changes, which were significantly higher in participants who accumulated > 150 min·wk^−1^ of moderate-to-vigorous intensity PA versus those who did not (13.06 (95% CI 7.51–18.61), *P* < 0.0001), whereas no relationship was detected for QoL.

**Conclusion:**

A counselling intervention that was effective in promoting a sustained change in PA and sedentary behaviour significantly improved psychological well-being and QoL.

**Trial Registration:**

ClinicalTrials.gov; NCT01600937; 10 October 2012.

**Supplementary Information:**

The online version contains supplementary material available at 10.1007/s40279-021-01556-0.

## Key Points

**Table:** 

In this randomized clinical trial that included 300 patients, an intervention that was effective in promoting a sustained change in physical activity and sedentary behaviour significantly improved scores of psychological well-being and physical and mental components of health-related quality of life.
The percentage of participants with (likely) depression decreased in the intervention group and increased in the control group.
Changes in psychological well-being and prevalence of depression, but not those in quality of life, were related to changes in physical activity volume.

## Introduction

Diabetes is known to be associated with depression, anxiety and other psychological distress or pathology as well as with reduced health-related quality of life (QoL). An average twofold increased prevalence of depression has in fact been reported in people with type 1 diabetes (T1D) and type 2 diabetes (T2D), based on either clinical diagnosis or self-report of depressive symptoms [1, 2]. Longitudinal prospective population-based studies have shown that this detrimental association is bi-directional. On the one hand, depression was found to confer an increased risk of incident diabetes [3, 4] by inducing diabetogenic health behaviours and possibly triggering common biological pathways [5], including increased counter-regulatory hormone release and action, alterations in glucose transport, and enhanced immunoinflammatory activation [6]. On the other hand, diabetes was shown to favour the development of depression [4], at least partly because of the psychological distress due to the burden of living with a demanding chronic disease and its disabling complications [7]. In addition, both diabetes and depression are associated to worse health scores [8] and reduced health-related QoL [9].

The dangerous association of depression (or depressive symptoms) and reduced QoL with diabetes is negatively linked to disease self-management through decreased compliance with diabetes monitoring and treatment [10, 11], ultimately resulting in poor glycaemic control [12] and increased likelihood to develop long-term diabetic complications [13]. This, in turn, may adversely affect psychological health [14, 15] and QoL [16], thus generating a vicious cycle resulting in worse disease outcomes. However, the relationship with diabetes self-management can also be the other way around, as recommended actions (e.g., glucose monitoring, insulin injections, exercise) and restrictions (e.g., dietary, sitting) may generate either negative or positive emotions that would limit or favour, respectively, adherence to disease monitoring and treatment, which is a critical issue in people with T2D [17] and involves all aspects, including compliance with physical activity (PA)/exercise and sedentary behaviour recommendations [18].

Two meta-analyses that investigated the effect of (mainly supervised) training programs of aerobic, resistance or combined exercise on QoL, symptoms of depression and anxiety, and emotional well-being found mixed results [19, 20]. However, in the Italian Diabetes and Exercise Study (IDES), we reported that physical and mental QoL scores improved with supervised aerobic and resistance exercise training [21] in a volume-dependent manner [22]. Compared to supervised exercise programs, counselling interventions targeting both PA/exercise and sedentary time (SED-time) may be more suitable for promoting a sustained behaviour change in T2D patients. Unfortunately, there are no data on whether these interventions are effective in improving well-being and QoL, thus favouring long-term maintenance of behaviour change.

In the IDES_2, we showed that a behavioural intervention compared with standard care resulted in a sustained increase in PA and decrease in SED-time over 3 years in individuals with T2D, associated with improvements in physical fitness and cardiovascular risk profile [23]. Here, we report on the effect of the intervention on psychological well-being and health-related QoL, which was a pre-specified secondary endpoint of this trial.

## Methods

The design and methods have been detailed elsewhere [23, 24] and are briefly reported here.

### Design

The IDES_2 was an open-label, assessor-blinded, parallel, superiority randomized clinical trial that assessed the efficacy of a behavioural intervention strategy in increasing PA and reducing SED-time in patients with T2D.

### Patients

The main entry criterion was T2D of ≥ 1-year duration. Additional requirements were: age 40–80 years; body mass index (BMI) 27–40 kg/m^2^; physical inactivity and sedentary lifestyle for at least 6 months; ability to walk 1.6 km without assistance; and eligibility after cardiologic evaluation.

### Randomization and Blinding

In three tertiary referral outpatient Diabetes Clinics in Rome, patients were randomized 1:1 to either an intervention (INT) group, receiving theoretical and practical exercise counselling, or a control (CON) group, receiving only general physician recommendations for increasing daily PA and decreasing SED-time.

Randomization was stratified by centre and, within each centre, by age (< 65 vs. ≥ 65 years) and diabetes treatment (non-insulin vs. insulin), using a permuted-block randomization SAS 9.4 software that randomly varies the block size (range 4–8).

Patients from both groups received the same treatment regimen, including dietary prescription, to achieve glycaemic, lipid, blood pressure (BP) and body-weight targets, according to current guidelines. Treatment was adjusted at each visit using a pre-specified algorithm.

Physicians, exercise specialists, and participants were not blinded, whereas assessors of PA/SED-time, biochemical parameters and well-being/QoL were blinded to group assignment.

### Intervention

The intervention in the INT group consisted of one individual theoretical counselling session, held by a diabetologist, plus eight twice-weekly individual theoretical and practical counselling sessions, held by a certified exercise specialist, once a year for 3 years (i.e., at months 1, 13 and 25).

### Outcome Measures

Co-primary endpoints were changes from baseline in total PA volume (metabolic equivalents (METs) h·wk^−1^), time spent in light-intensity PA (LPA, h·day^−1^) and moderate-to-vigorous-intensity PA (MVPA, min·day^−1^), and SED-time (h·day^−1^) over the 3-year period. Secondary endpoints were changes from baseline in physical fitness, modifiable cardiovascular factors and scores, and well-being/QoL. Here, we report on the effect of intervention on psychological well-being and health-related QoL, whereas results for the other endpoints have been presented in a previous publication [23].

### Psychological Well-Being and Health-Related Quality of Life (QoL)

At baseline and at 4, 12, 16, 24, 28 and 36 months of follow-up, psychological well-being and health-related QoL were assessed using the World Health Organization-5 (WHO-5) Well-Being Index and the 36-Item Short Form Health Survey (SF-36), respectively.

The WHO-5 Well-Being Index is a five-item questionnaire developed from the WHO-10 Well-Being Index, which is suitable for use in patients with T1D and T2D [25]. Each of the five positively worded items is scored on a six-point Likert scale ranging from 0 to 5 and is transformed to a score from 0 (worst) to 100 (best). The scale has adequate validity both as an outcome measure in clinical trials and as a screening tool for depression, with a score of ≤ 50 suggesting suboptimal well-being and the need for further testing and a score ≤ 28 representing likely depression [26].

The SF-36, which was previously validated in patients with T2D [27], is a 36-item multipurpose survey that evaluates eight fields of life, which are further grouped into the physical component summary (PCS) and the mental component summary (MCS) [28]. These aggregated scores are converted into norm-based scores (mean, 50; SD, 10), with higher scores indicating a more favourable physical functioning and psychological well-being [28].

### Other Measurements

At baseline and every 4 months thereafter, PA volume, LPA, MVPA and SED-time were measured using an accelerometer (MyWellness Key, Technogym, Cesena, Italy) and a daily diary for non-accelerometer recordable activities.

At the same time points, the modifiable cardiovascular risk factors BMI, waist circumference, fasting plasma glucose; glycated haemoglobin (HbA_1c_); triglycerides; total, HDL and LDL cholesterol; systolic and diastolic BP; and high-sensitivity C-reactive protein were assessed using standard methods, and coronary heart disease (CHD) and stroke 10-year risk scores were calculated using the United Kingdom Prospective Diabetes Study (UKPDS) risk engine.

At baseline and every year thereafter, participants were evaluated for physical fitness by assessing cardiorespiratory fitness (as maximal oxygen uptake, *V*O_2max_), strength and flexibility by maximal treadmill exercise test, isometric test and bending test, respectively.

### Statistical Analysis

Sample size calculation for the primary endpoint has been reported elsewhere [23].

The nonparametric Mann–Whitney test was utilized for between-group comparisons of baseline measures, as appropriate. Frequency (*n*) and percentage (%) of participants in the two groups presenting at each time point with the WHO-5 score indicating optimal or suboptimal well-being or likely depression were compared using Pearson’s *χ*^2^ test.

The superiority of the intervention versus standard care on psychological well-being and health-related QoL was assessed by mixed models for repeated measures in the whole cohort and in participants stratified by age (< 65 and ≥ 65 years) and gender. The overall estimated scores for WHO-5, PCS, MCS and individual SF-36 domains during the 3-year follow-up were assessed by contrasts generated from mixed models, and results are expressed as estimated mean difference and 95% Wald confidence interval (CI). Models for repeated measures with an autoregressive correlation type matrix make an assumption of missing at random and account for both missingness at random and potential correlation within participants, as they allow evaluating all individuals, including those with incomplete data [23]. In addition, score changes detected in the 50 individuals (49 from the INT and one from the CON group) who became physically active, i.e., accumulated ≥ 150 min·wk^−1^ of MVPA throughout the 3-year period, were compared with those in the remaining 250 participants by mixed models for repeated measures.

Linear regression analyses were applied to assess the independent correlates of baseline to end-of-study changes in WHO-5, PCS and MCS scores. Age, gender, baseline value of each score and either baseline levels (Model 1) or baseline to end-of-study changes (Model 2) of BMI, HbA_1c_, systolic BP and either physical fitness (*V*O_2max_ and lower body muscle strength) or activity (PA volume and SED-time) parameters were included as covariates in the regression models. Logistic regression analysis was applied to assess the independent correlates of likely depression (WHO-5 scores ≤ 28) using the covariates indicated above.

All *P* values < 0.05 were considered statistically significant. Statistical analyses were performed using SAS software release 9.4 (Cary, NC, USA) and R version 4.0.0.

## Results

**Fig. 1:**
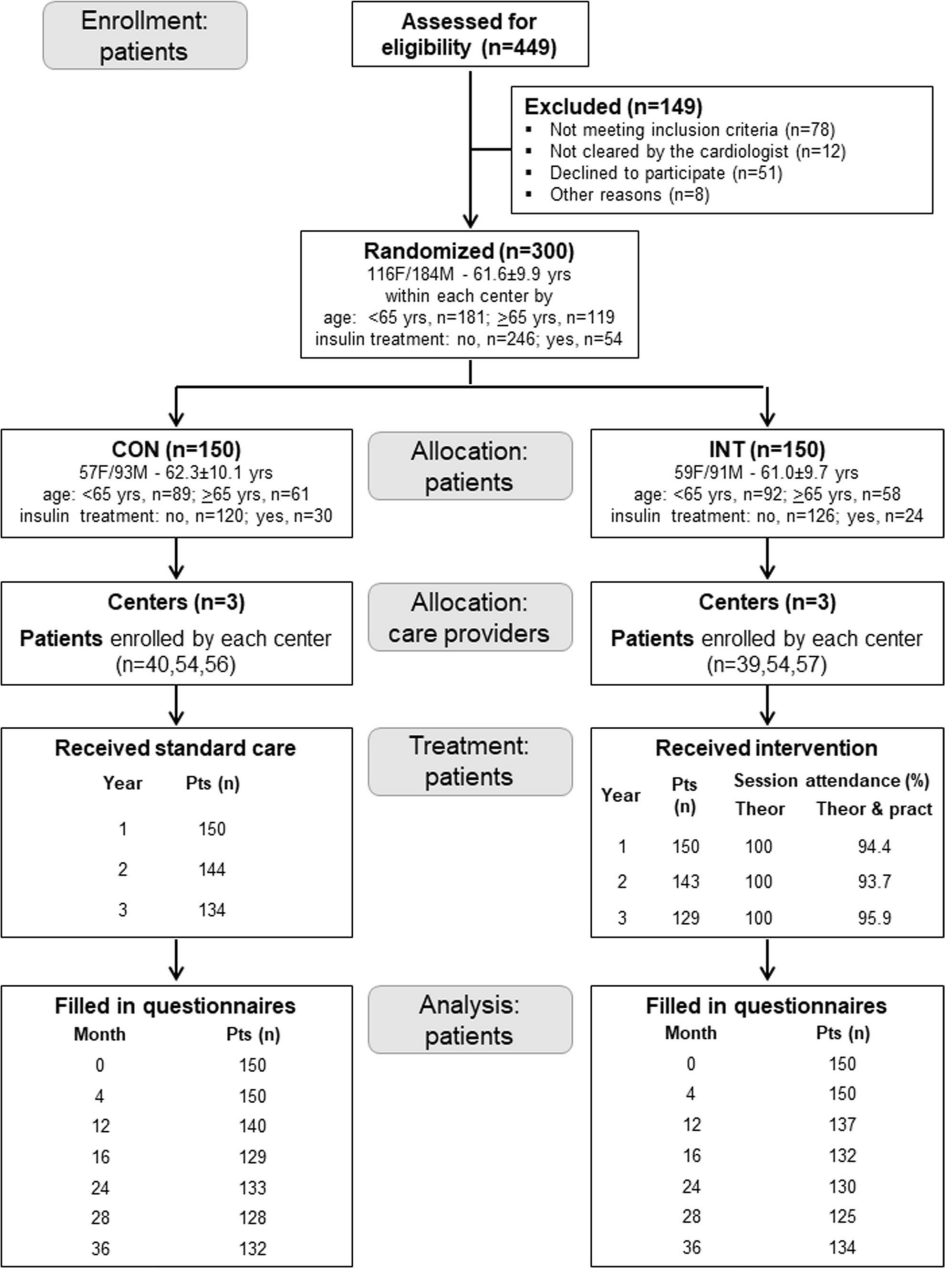
Study flow chart. Flow of participants through the study. *CON* control group, *INT* intervention group, *F* females, *M* males

A total of 449 patients were assessed for eligibility from October 2012 to February 2014 in the three centres and, after excluding 149 patients for various reasons, the remaining 300 were randomized to CON or INT group (*n* = 150 each) (Fig. 1). The baseline features of study participants have been reported elsewhere [23].

As previously reported [23], the percentages of session attendance and study completion were > 90% and 89%, respectively, and adverse events did not differ between groups and were unrelated to PA. The number of participants who filled in the questionnaires is shown in Fig. 1.

### Co-Primary and Other Secondary Outcomes

The main trial outcomes have been reported elsewhere [23]. Briefly, as compared with CON, INT participants showed an increase over time in PA volume, MVPA and LPA, and a reciprocal decrease in SED-time. Significant between-group differences were maintained throughout the study, though difference in MVPA diminished ~ twofold during the third year. These changes were associated with sustained improvements in physical fitness, including *V*O_2max_, lower body strength and flexibility, and modifiable cardiovascular risk factors/scores, including fasting plasma glucose, systolic BP, and total and fatal CHD 10-year risk scores, in INT versus CON participants.

### Psychological Well-Being

**Fig. 2:**
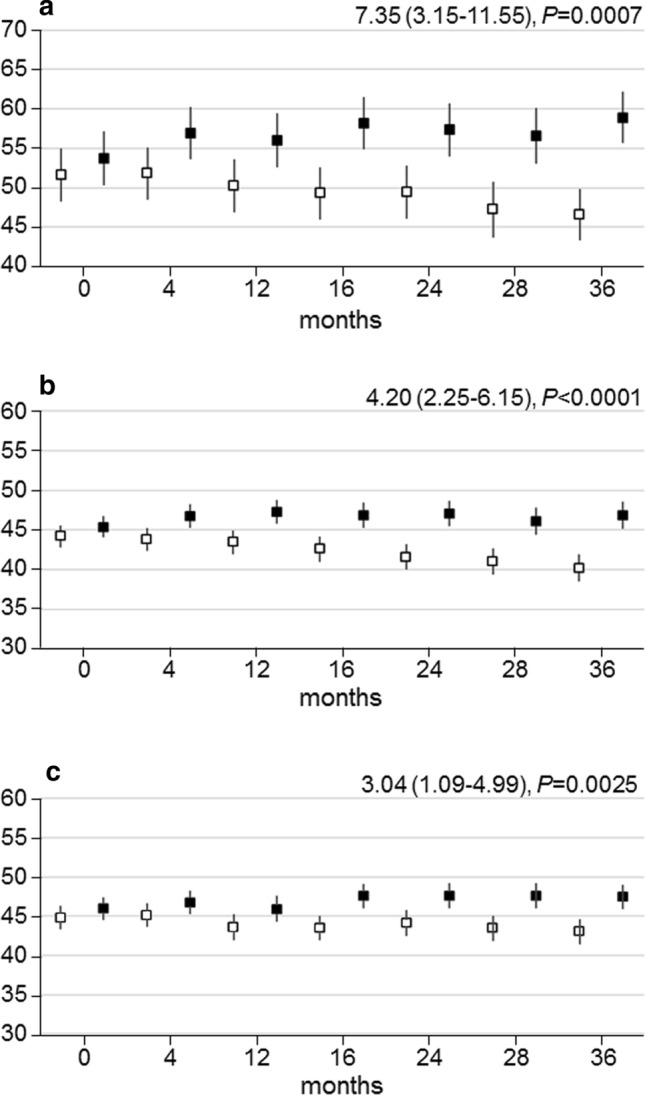
WHO-5, PCS and MCS scores over time. Change over time in WHO-5 (**a**), PCS (**b**) and MCS (**c**) scores in participants in the INT (black boxes) versus CON (white boxes) group. Data [estimated mean with 95% confidence interval (CI)] were calculated on the basis of questionnaires filled in at scheduled visits. The analyses are based on a mixed model for repeated measurements, taking into account within-participant correlation. *P* values were calculated with a mixed model for repeated measurements. Estimated mean differences over time with 95% CIs and *P* values between INT and CON are reported at the top right. *WHO* World Health Organization, *PCS* physical component summary, *MCS* mental component summary, *CON* control group, *INT* intervention group

The WHO-5 scores did not differ between the two groups at baseline (Table 1); thereafter, they increased in the INT and decreased in the CON participants (Fig. 2a). Over the 3-year period, participants in the INT group perceived a significantly higher state of psychological well-being compared to those in the CON group (7.35 (95% CI 3.15–11.55), *P* = 0.0007) (Fig. 2a). Between-group differences over time were similar in participants aged < 65 and ≥ 65 years (Online Supplementary Material (OSM) Fig. 1a, b); conversely, though the trend was similar in the two sexes, differences between INT and CON participants were significant only in females, who showed much lower baseline WHO scores (OSM Fig. 1c, d).

**Table 1:** Baseline values of WHO-5 and SF-36 scores by study arm

Variable	CONT	INT	P*
WHO-5	51.6 ± 21.9	53.7 ± 20.4	0.48
SF-36			
Physical functioning	46.3 ± 9.2	46.9 ± 8.4	0.72
Limitation physical	47.1 ± 10.3	47.2 ± 9.9	0.90
Bodily pain	43.8 ± 10.8	46.2 ± 10.4	0.05
General health	38.4 ± 10.9	39.8 ± 9.3	0.33
Emotional/mental health	41.6 ± 11.0	43.8 ± 10.5	0.08
Limitation emotional	50.7 ± 8.9	49.5 ± 10.2	0.50
Social functioning	40.8 ± 9.7	43.0 ± 9.0	0.04
Energy/vitality	46.1 ± 9.6	47.7 ± 8.6	0.28
PCS	44.2 ± 9.1	45.4 ± 8.0	0.29
MCS	44.8 ± 9.0	45.9 ± 9.4	0.25

Values are mean ± SD

*WHO* World Health Organization, *SF-36* 36-Item Short Form, *PCS* physical component summary, *MCS* mental component summary, *CON* control group, *INT* intervention group

*Mann–Whitney *U* test

The percentage of participants with likely depression decreased in the INT group and increased in the CON group during the 3-year period, with statistically significant between-group differences after 1 year from randomization (Table 2). Again, the effect of intervention was similar in participants aged < 65 and ≥ 65 years (OSM Table 1) and lower in males than in females, who showed a much greater prevalence of likely depression and, to a lesser extent, of sub-optimal well-being (OSM Table 2).

**Table 2:** Percentage of participants with optimal and suboptimal psychological well-being and likely depression in the whole cohort and by study arm

	Months						
	0	4	12	16	24	28	36
Optimal							
Total	174 (58.0)	181 (60.3)	164 (59.2)	153 (58.6)	158 (60.1)	147 (58.1)	159 (59.8)
CON	85 (56.7)	85 (56.7)	74 (52.9)	60 (46.5)	71 (53.4)	61 (47.7)	63 (47.7)
INT	89 (59.3)	96 (64.0)	90 (65.7)	93 (70.5)	87 (66.9)	86 (68.8)	96 (71.6)
Suboptimal							
Total	75 (25.0)	76 (25.3)	67 (24.2)	64 (24.5)	56 (21.3)	58 (22.9)	62 (23.3)
CON	36 (24.0)	39 (26.0)	38 (27.1)	39 (30.2)	30 (22.6)	35 (27.3)	36 (27.3)
INT	39 (26.0)	37 (24.7)	29 (21.2)	25 (18.9)	26 (20.0)	23 (18.4)	26 (19.4)
Likely depression							
Total	51 (17.0)	43 (14.3)	46 (16.6)	44 (16.9)	49 (18.6)	48 (19.0)	45 (16.9)
CON	29 (19.3)	26 (17.3)	28 (20.0)	30 (23.3)	32 (24.1)	32 (25.0)	33 (25.0)
INT	22 (14.7)	17 (11.3)	18 (13.1)	14 (10.6)	17 (13.1)	16 (12.8)	12 (9.0)
*N*	300	300	277	261	263	253	266
*p* CON vs INT*	0.556	0.272	0.086	< 0.0001	0.039	0.002	< 0.0001

Values are *n* (%)

*CON* control group, *INT* intervention group

*Pearson’s *χ*^2^ test

**Fig. 3:**
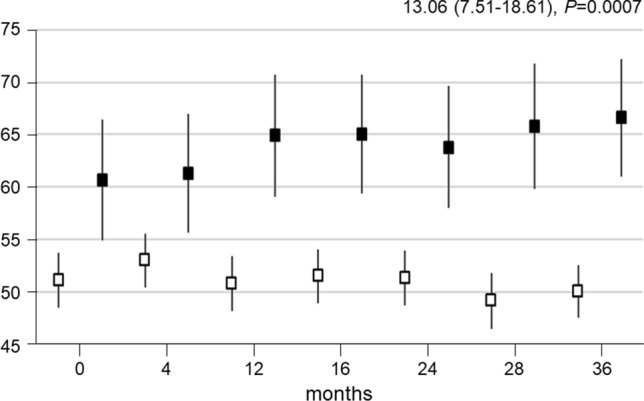
WHO-5 scores over time by physically active status. Change over time in WHO-5 scores in participants accumulating (black boxes) and not accumulating (white boxes) 150 min·wk^−1^ of MVPA. Data (estimated mean with 95% CI) were calculated on the basis of questionnaires filled in at scheduled visits. The analyses are based on a mixed model for repeated measurements, taking into account within-participant correlation. *P* values were calculated with a mixed model for repeated measurements. Estimated mean differences over time with 95% CIs and *P* values between INT and CON are reported at the top right. *WHO* World Health Organization, *CON* control group, *INT* intervention group

The 50 participants who reached or exceeded the recommended MVPA level of 150 min·wk^−1^ over the 3-year follow-up perceived a higher state of psychological well-being compared to those who did not (13.06 (95% CI 7.51–18.61), *P* < 0.0001); this difference became statistically significant after 28 months from randomization (Fig. 3).

Higher baseline WHO-5 value, *V*O_2max_, PA volume and SED-time as well as baseline to end-of-study changes in *V*O_2max_, lower body strength and PA volume were independent predictors of higher baseline to end-of-study changes in WHO-5 scores; most of these variables were also independent predictors of likely depression (OSM Table 3).

### QoL

**Fig. 4:**
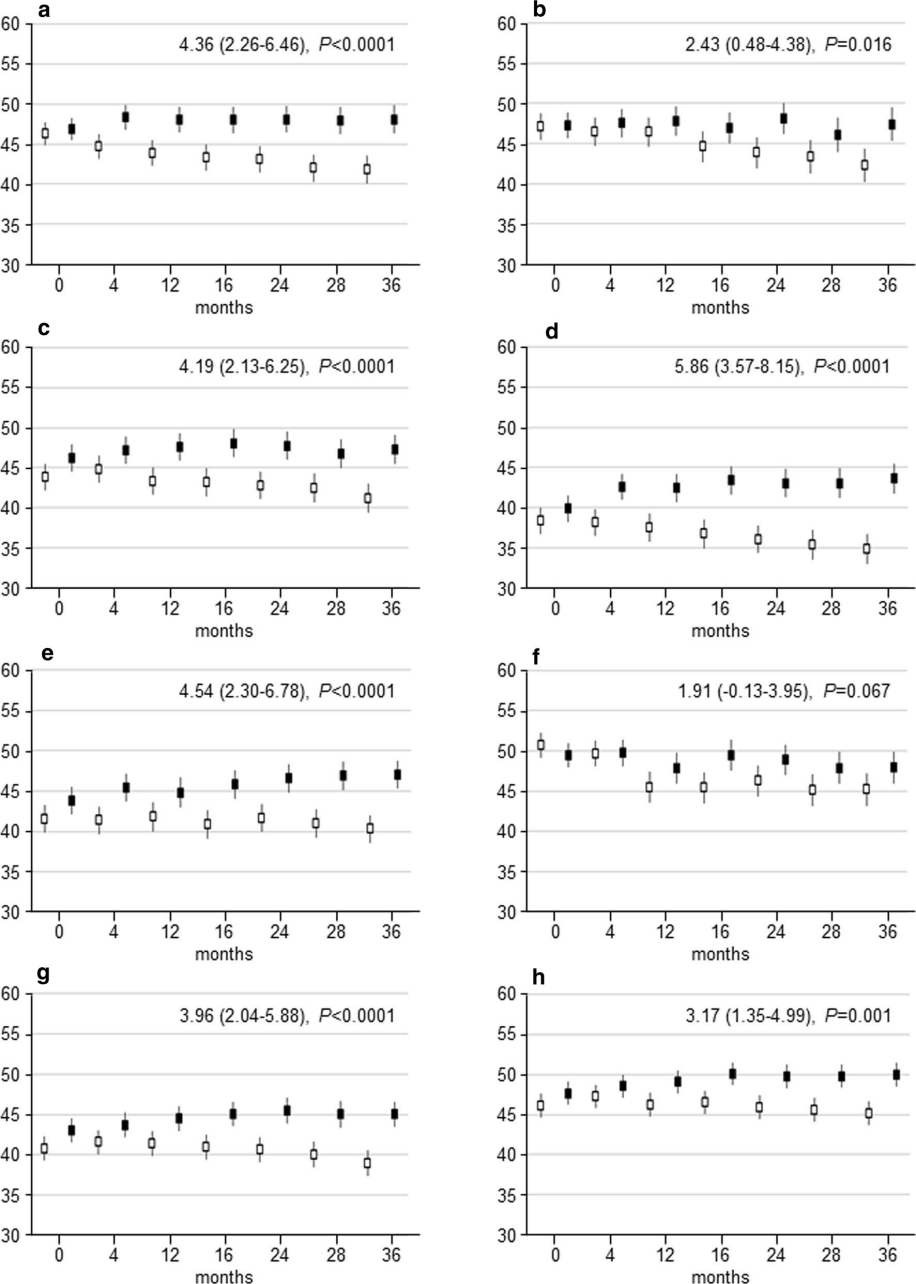
Physical and mental domain scores over time. Change over time in SF-36 scores in physical functioning (**a**), limitation physical (**b**), bodily pain (**c**), general health (**d**), emotional/mental health (**e**), limitation emotional (**f**), social functioning (**g**) and energy/vitality (**h**) in participants in the INT (black boxes) versus CON (white boxes) group. Data [estimated mean with 95% confidence interval (CI)] were calculated on the basis of questionnaires filled in at scheduled visits. The analyses are based on a mixed model for repeated measurements, taking into account within-participant correlation. *P* values were calculated with a mixed model for repeated measurements. Estimated mean differences over time with 95% CIs and *P* values between INT and CON are reported at the top right. *SF-36* 36-Item Short Form, *CON* control group, *INT* intervention group

Baseline SF-36 scores did not differ between the two groups, except for a slightly higher social functioning score in the INT versus CON participants (Table 1). Gradual and sustained improvements in PCS and MCS were observed in the INT group, whereas these scores worsened progressively in the CON group, resulting in significant between-group differences over the 3-year period (PCS: 4.20 (95% CI 2.25–6.15), *P* < 0.0001; MCS: 3.04 (95% CI 1.09–4.99), *P* = 0.0025) (Fig. 2b, c). Significant differences were also detected in all physical and mental domains except for the domain score ‘limitation emotional’ (Fig. 4). The effect of intervention on PCS and MCS was similar in participants aged < 65 years and those aged ≥ 65 years and in males and females, though between-group differences over time were not significant in older individuals and females for MCS (OSM Fig. 2).

No statistically significant differences were found between participants accumulating or not accumulating ≥ 150 min·wk^−1^ of MVPA throughout the 3-year period (not shown).

Higher baseline PCS and MCS values were independent predictors of higher baseline to end-of-study changes in PCS and MCS scores, respectively; in addition, higher baseline BMI and baseline to end-of-study changes in HbA_1c_ were independent predictors of higher PCS and MCS scores, respectively (OSM Table 4).

## Discussion

Psychological well-being and QoL are considered important treatment goals in people with diabetes, in addition to traditional medical outcomes [29]. Moreover, they represent main factors for achieving optimal diabetes self-management [10, 11], including the adoption and maintenance of an active lifestyle, which in turn might affect psychological factors [19, 20].

### Effects of Intervention on Well-Being/QoL

We have shown that a behavioural intervention, which was successful in promoting a sustained increase in PA and decrease in SED-time, was associated with a progressive improvement in psychological well-being and health-related QoL and reduction in the number of participants with likely depression, whereas opposite trends were observed in the CON group.

These findings add substantially to our understanding of the relationships between PA/exercise and well-being/QoL. In fact, prior publications reported almost invariably on exercise intervention studies in which participants were engaged in aerobic and/or resistance training sessions in gym facilities under the supervision of exercise specialists. In the IDES, we found a positive effect of a supervised aerobic and resistance training program of 1 year’s duration on all components of health-related QoL [21]. Likewise, a 2-year multicomponent exercise intervention showed improved physical and mental health-related QoL in older adults with T2D [30]. However, two meta-analyses reported mixed results, because many other studies failed to detect a positive effect of supervised exercise programs [19, 20]. These contrasting findings might be related to the reported U-shaped relationship between diabetes-specific health behaviours, including diet and exercise, and perceived burden [31], i.e., after a certain threshold, the more demanding the lifestyle program, the greater the burden patients perceive. Conversely, in the IDES_2, participants received a 1-month counselling intervention each year aimed at promoting a behaviour change during the remaining 11 months by encouraging them to perform PA on their own and to reduce (and interrupt) SED-time as much as possible. This approach could have been less burdensome as it resulted in less striking increases in PA, especially MVPA, and, therefore, it may have had a more favourable impact on QoL, compared to some, more demanding, supervised exercise programs. The counselling intervention of the IDES_2 was in fact effective in improving psychological well-being and health-related QoL and, in turn, this positive impact on psychosocial factors may have contributed to maintenance of behaviour change in the long term.

The intervention was also effective in reducing the prevalence of likely depression among the study participants, consistent with an isotemporal substitution study showing that replacing even small amounts of SED-time with LPA may reduce depression in older adults [32].

Of note, the effects of the behavioural counselling on well-being and QoL were even more striking when considering that both deteriorated in the CON group, consistent with the known worsening of these scores associated with diabetes [4, 8, 9]. Furthermore, as already known [33, 34], prevalence of likely depression was much higher in females than in males, a finding that may explain why the opposite effects of intervention and standard care on WHO scores were more pronounced in women than in men.

### Predictors of Well-Being/QoL Changes

An intriguing finding is that the effect of the intervention on psychological well-being was related to increases in PA volume and achievement of a physically active status, whereas that on QoL was not. Changes in PA volume (and related changes in fitness parameters) were in fact independently associated with changes in WHO-5 scores, which were significantly higher in participants who accumulated ≥ 150 min·wk^−1^ of MVPA over the 3-year follow-up, compared to those who did not. Conversely, no relationships with PA/sedentary behaviour or fitness parameters were detected for health-related QoL. This finding is in contrast with our previous IDES report in T2D patients engaged in a 1-year supervised exercise program [22]. It is also at odds with two cross-sectional surveys in older adults reporting higher physical QoL scores in individuals meeting MVPA recommendations compared to those who did not [35] and significantly better PCS if replacing SED-time or LPA with MVPA [36]. In the present study, the intervention per se seems to be important for the improvements in QoL measures, regardless of its efficacy in terms of increase in PA volume (and decrease in SED-time). This interpretation is consistent with a previous report in individuals with knee and hip osteoarthritis participating in an educational exercise program, who showed increased QoL despite no change in PA and SED-time [37]. However, our finding might also reflect the U-shaped relationship between PA and perceived burden of adherence to lifestyle recommendations [31], with individuals maintaining a more active and less sedentary lifestyle for such a long period of time showing a negative (or less positive) impact on QoL compared with those who responded to the intervention with a more modest change in behaviour.

The independent associations of changes in PCS with baseline BMI and of changes in MCS with changes in HbA_1c_ are difficult to explain as they seem to suggest that higher body weight at the start and deterioration of glycaemic control during the study impacted favourably on QoL. Moreover, the finding that well-being and QoL changes were related to baseline scores supports the concept that prior psychosocial status is a major determinant of the impact of diabetes burden, including implementation of lifestyle measures, on perception of physical and mental health.

### Strengths and Limitations

The main strengths of this study concern the application of an intervention targeting both PA and SED-time, the specific training of investigators, the long study duration, and the large sample size. Other strengths include the use of validated questionnaires for assessing psychological well-being and health-related QoL, together with objective (accelerometer-based) measurement of PA/SED-time and concurrent assessment of physical fitness and cardiovascular risk profile.

This study has several limitations. First, generalizability requires further investigation and validation in different cohorts or settings. Second, as the WHO-5 Well-Being Index is only a screening tool for depression, which requires additional tests for diagnosis and classification [38], results regarding the impact of intervention on the prevalence of depression should be interpreted as exploratory. Third, results might have been affected by unmeasured confounders, for instance diet, that were not considered in the data analysis, though patients received dietary prescriptions and adherence to diet was verified at intermediate visits.

## Conclusion

A counselling intervention that was effective in producing a sustained behavioural change with increased PA and reduced SED-time significantly improved psychological well-being and health-related QoL and decreased the proportion of participants with likely depression, compared with standard care, which was associated with worsening of all scores. Changes in psychological well-being and prevalence of likely depression, but not those in PCS and MCS, were related to changes in PA volume.

## Supplementary Information

Below is the link to the electronic supplementary material.

Supplementary file 1 (DOCX 1013 kb)

## Abbreviations

QoL: Quality of life
PA: Physical activity
IDES: Italian Diabetes and Exercise Study
SED-time: Sedentary time
INT: Intervention
CON: Control
BMI: Body mass index
BP: Blood pressure
LPA: Light-intensity PA
MVPA: Moderate-to-vigorous-intensity PA
METs: Metabolic equivalents
HbA_1c_: Glycated haemoglobin
CHD: Coronary heart disease
UKPDS: United Kingdom Prospective Diabetes Study
*V*O_2max_: Maximal oxygen uptake
WHO: World Health Organization
SF-36: 36-Item short form
PCS: Physical component summary
MCS: Mental component summary
CHD: Coronary heart disease
UKPDS: United Kingdom Prospective Diabetes Study
CI: Confidence interval

## Declarations

### Data availability statement

The datasets generated during and/or analysed during the current study are available from the corresponding author on reasonable request.
