# Supplementary material for: Effect of a Behavioural Intervention for Adoption and Maintenance of a Physically Active Lifestyle on Psychological Well-Being and Quality of Life in Patients with Type 2 Diabetes: The IDES_2 Randomized Clinical Trial
Source: Sports Med. 2021 Oct 1;52(3):643–54. doi: 10.1007/s40279-021-01556-0 (PMC8891112; doi:10.1007/s40279-021-01556-0)
Supplement: Supplementary file 1 — (DOCX 1013 kb) [file 40279_2021_1556_MOESM1_ESM.docx]

**Effect of a behavioural intervention for adoption and maintenance of a physically active lifestyle on psychological well-being and quality of life in patients with type 2 diabetes: The IDES_2 randomized clinical trial.**

Antonio Nicolucci, Jonida Haxhi, Valeria D’Errico, Massimo Sacchetti, Giorgio Orlando, Patrizia Cardelli, Martina Vitale, Lucilla Bollanti, Francesco Conti, Silvano Zanuso, Giuseppe Lucisano, Stefano Balducci, and Giuseppe Pugliese*, for the Italian Diabetes and Exercise Study 2 (IDES_2) Investigators.

**Corresponding Author:** University of Rome La Sapienza, Rome, Italy; [giuseppe.pugliese@uniroma1.it](mailto:giuseppe.pugliese@uniroma1.it).

**The IDES_2 Investigators**

**ESM Table 1** Percentage of participants aged < or > 65 years with optimal and suboptimal psychological well-being and likely depression, total and by study arm.

**ESM Table 2** Percentage of male and female participants with optimal and suboptimal psychological well-being and likely depression, total and by study arm.

**ESM Table 3** Independent predictors of baseline to end-of-study changes in WHO-5 score and WHO-5 score <28 (likely depression).

**ESM Table 4** Independent predictors of baseline to end-of-study changes in PCS and MCS score.

**ESM Fig 1** WHO-5 scores over time by age and gender.

**ESM Fig 2** PCS and MCS scores over time by age and gender.

**The IDES_2 Investigators**

**Steering Committee:**

Giuseppe Pugliese, MD, PhD, Department of Clinical and Molecular Medicine, University of Rome La Sapienza, and Diabetes Unit, Sant’Andrea University Hospital, Rome, Italy (Principal Investigator);

Stefano Balducci, MD, Metabolic Fitness Association O.N.L.U.S., Monterotondo, Rome, Italy (Co-Investigator);

Massimo Sacchetti, PhD, Department of Human Movement and Sport Sciences, University of Rome ‘Foro Italico’, Rome, Italy;

Silvano Zanuso, PhD, Centre for Applied Biological & Exercise Sciences, Faculty of Health & Life Sciences, Coventry University, Coventry, United Kingdom;

Patrizia Cardelli, PhD, Department of Clinical and Molecular Medicine, University of Rome La Sapienza, and Laboratory of Clinical Chemistry, Sant’Andrea University Hospital, Rome, Italy;

Antonio Nicolucci, MD, PhD, Centre for Outcomes Research and Clinical Epidemiology (CORESEARCH), Pescara, Italy.

**Participating centers:**

***Diabetes Clinics***

1. Diabetes Unit, Sant’Andrea University Hospital, Rome, Italy: Giuseppe Pugliese, MD, PhD, Maria Cristina Ribaudo, MD, PhD, Elena Alessi, MD, Martina Vitale, MD, Tiziana Cirrito, MD, Lucilla Bollanti, MD, Francesco G. Conti, MD, PhD.
2. Diabetes Unit, Fatebenefratelli San Pietro Hospital, Rome, Italy: Nicolina Di Biase, MD, Filomena La Saracina, MD.
3. Diabetes Unit, Health District, Monterotondo, Rome, Italy: Stefano Balducci, MD, Mario Ranuzzi, MD, Jonida Haxhi, MD, PhD, Valeria D’Errico, MD.

***Metabolic Fitness Centres***

1. Department of Human Movement and Sport Sciences, University of Rome ‘Foro Italico’, Rome, Italy: Massimo Sacchetti, PhD, Giorgio Orlando, PhD.
2. Centre for the Study of Metabolism, Rome, Italy: Luca Milo, Physiotherapist, Roberto Milo, Physiotherapist.
3. Metabolic Fitness Association, Monterotondo, Rome, Italy: Gianluca Balducci, Physiotherapist, Enza Spinelli, Physiotherapist.

**ESM Table 1** Percentage of participants aged < or > 65 years with optimal and suboptimal psychological well-being and likely depression, total and by study arm.

|  | **Months** | | | | | | |
| --- | --- | --- | --- | --- | --- | --- | --- |
|  | **0** | **4** | **12** | **16** | **24** | **28** | **36** |
| **Aged <65 years** |  |  |  |  |  |  |  |
| **Optimal** |  |  |  |  |  |  |  |
| **Total** | 105 (58.0) | 106 (58.6) | 93 (57.4) | 83 (55.3) | 93 (60.8) | 83 (56.1) | 99 (63.5) |
| **CON** | 52 (58.4) | 52 (58.4) | 43 (52.4) | 33 (44.6) | 44 (56.4) | 37 (48.7) | 41 (53.9) |
| **INT** | 53 (57.6) | 54 (58.7) | 50 (62.5) | 50 (65.8) | 49 (65.3) | 46 (63.9) | 58 (72.5) |
| **Suboptimal** |  |  |  |  |  |  |  |
| **Total** | 50 (27.6) | 52 (28.7) | 45 (27.8) | 43 (28.7) | 36 (23.5) | 41 (27.7) | 38 (24.4) |
| **CON** | 21 (23.6) | 23 (25.8) | 22 (26.8) | 25 (33.8) | 18 (23.1) | 21 (27.6) | 21 (27.6) |
| **INT** | 29 (31.5) | 29 (31.5) | 23 (28.8) | 18 (23.7) | 18 (24.0) | 20 (27.8) | 17 (21.3) |
| **Likely depression** |  |  |  |  |  |  |  |
| **Total** | 26 (14.4) | 23 (12.7) | 24 (14.8) | 24 (16.0) | 24 (15.7) | 24 (16.2) | 19 (12.2) |
| **CON** | 16 (18.0) | 14 (15.7) | 17 (20.7) | 16 (21.6) | 16 (20.5) | 18 (23.7) | 14 (18.4) |
| **INT** | 10 (10.9) | 9 (9.8) | 7 (8.8) | 8 (10.5) | 8 (10.7) | 6 (8.3) | 5 (6.3) |
| **N** | 181 | 181 | 162 | 150 | 153 | 148 | 156 |
| ***p* CON vs INT*** | 0.269 | 0.413 | 0.096 | 0.026 | 0.237 | 0.032 | 0.023 |
| **Aged >65 years** |  |  |  |  |  |  |  |
| **Optimal** |  |  |  |  |  |  |  |
| **Total** | 69 (58.0) | 75 (63.0) | 71 (61.7) | 70 (63.1) | 65 (59.1) | 64 (61.0) | 60 (54.5) |
| **CON** | 33 (54.1) | 33 (54.1) | 31 (53.4) | 27 (49.1) | 27 (49.1) | 24 (46.2) | 22 (39.3) |
| **INT** | 36 (62.1) | 42 (72.4) | 40 (70.2) | 43 (76.8) | 38 (69.1) | 40 (75.5) | 38 (70.4) |
| **Suboptimal** |  |  |  |  |  |  |  |
| **Total** | 25 (21.0) | 24 (20.2) | 22 (19.1) | 21 (18.9) | 20 (18.2) | 17 (16.2) | 24 (21.8) |
| **CON** | 15 (24.6) | 16 (26.2) | 16 (27.6) | 14 (25.5) | 12 (21.8) | 14 (26.9) | 15 (26.8) |
| **INT** | 10 (17.2) | 8 (13.8) | 6 (10.5) | 7 (12.5) | 8 (14.5) | 3 (5.7) | 9 (16.7) |
| **Likely depression** |  |  |  |  |  |  |  |
| **Total** | 25 (21.0) | 20 (16.8) | 22 (19.1) | 20 (18.0) | 25 (22.7) | 24 (22.9) | 26 (23.6) |
| **CON** | 13 (21.3) | 12 (19.7) | 11 (19.0) | 14 (25.5) | 16 (29.1) | 14 (26.9) | 19 (33.9) |
| **INT** | 12 (20.7) | 8 (13.8) | 11 (19.3) | 6 (10.7) | 9 (16.4) | 10 (18.9) | 7 (13.0) |
| **N** | 119 | 119 | 115 | 111 | 110 | 105 | 110 |
| ***p* CON vs INT*** | 0.578 | 0.107 | 0.058 | 0.010 | 0.099 | 0.003 | 0.004 |
| ***p* < vs >65 years *** | 0.210 | 0.206 | 0.218 | 0.194 | 0.274 | 0.073 | 0.049 |

Values are n (%). * Pearson’s χ^2^ test. CON = control group; INT = intervention group.

**ESM Table 2** Percentage of male and female participants with optimal and suboptimal psychological well-being and likely depression, total and by study arm.

|  | **Months** | | | | | | |
| --- | --- | --- | --- | --- | --- | --- | --- |
|  | **0** | **4** | **12** | **16** | **24** | **28** | **36** |
| **Males** |  |  |  |  |  |  |  |
| **Optimal** |  |  |  |  |  |  |  |
| **Total** | 132 (71.4) | 131 (70.8) | 125 (72.7) | 105 (67.7) | 114 (70.8) | 109 (70.8) | 113 (68.5) |
| **CON** | 66 (70.2) | 66 (70.2) | 61 (68.5) | 45 (58.4) | 56 (67.5) | 49 (62.0) | 50 (60.2) |
| **INT** | 66 (72.5) | 65 (71.4) | 64 (77.1) | 60 (76.9) | 68 (74.4) | 60 (80.0) | 63 (76.8) |
| **Suboptimal** |  |  |  |  |  |  |  |
| **Total** | 37 (20.0) | 38 (20.5) | 35 (20.3) | 34 (21.9) | 31 (19.3) | 31 (20.1) | 36 (21.8) |
| **CON** | 19 (20.2) | 21 (22.3) | 20 (22.5) | 23 (29.9) | 18 (21.7) | 22 (27.8) | 22 (26.5) |
| **INT** | 18 (19.8) | 17 (18.7) | 15 (18.1) | 11 (14.1) | 13 (16.7) | 9 (12.0) | 14 (17.1) |
| **Likely depression** |  |  |  |  |  |  |  |
| **Total** | 16 (8.6) | 16 (8.6) | 12 (7.0) | 16 (10.3) | 16 (9.9) | 14 (9.1) | 16 (9.7) |
| **CON** | 9 (9.6) | 7 (7.4) | 8 (9.0) | 9 (11.7) | 9 (10.8) | 8 (10.1) | 11 (13.3) |
| **INT** | 7 (7.7) | 9 (9.9) | 4 (4.8) | 7 (9.0) | 7 (9.0) | 6 (8.0) | 5 (6.1) |
| **N** | 185 | 185 | 172 | 155 | 161 | 154 | 165 |
| ***p* CON vs INT*** | 0.892 | 0.730 | 0.384 | 0.036 | 0.626 | 0.034 | 0.063 |
| **Females** |  |  |  |  |  |  |  |
| **Optimal** |  |  |  |  |  |  |  |
| **Total** | 42 (36.5) | 50 (43.5) | 39 (37.1) | 48 (45.3) | 44 (43.1) | 38 (38.4) | 46 (45.5) |
| **CON** | 19 (33.9) | 19 (33.9) | 13 (25.5) | 15 (28.8) | 15 (30.0) | 12 (24.5) | 13 (26.5) |
| **INT** | 23 (39.0) | 31 (52.5) | 26 (48.1) | 33 (61.1) | 29 (55.8) | 26 (52.0) | 33 (63.5) |
| **Suboptimal** |  |  |  |  |  |  |  |
| **Total** | 38 (33.0) | 38 (33.0) | 32 (30.5) | 30 (28.3) | 25 (24.5) | 27 (27.3) | 26 (25.7) |
| **CON** | 17 (30.4) | 18 (32.1) | 18 (35.3) | 16 (30.8) | 12 (24.0) | 13 (26.5) | 14 (28.6) |
| **INT** | 21 (35.6) | 20 (33.9) | 14 (25.9) | 14 (25.9) | 13 (25.0) | 14 (28.0) | 12 (23.1) |
| **Likely depression** |  |  |  |  |  |  |  |
| **Total** | 35 (30.4) | 27 (23.5) | 34 (32.4) | 28 (26.4) | 33 (32.4) | 34 (34.3) | 29 (28.7) |
| **CON** | 20 (35.7) | 19 (33.9) | 20 (39.2) | 21 (40.4) | 23 (46.0) | 24 (49.0) | 22 (44.9) |
| **INT** | 15 (25.4) | 8 (13.6) | 14 (25.9) | 7 (13.0) | 10 (19.2) | 10 (20.0) | 7 (13.5) |
| **N** | 115 | 115 | 105 | 106 | 102 | 99 | 101 |
| ***p* CON vs INT*** | 0.487 | 0.025 | 0.055 | 0.001 | 0.008 | 0.004 | <0.0001 |
| ***p* males vs females*** | <0.0001 | <0.0001 | <0.0001 | <0.0001 | <0.0001 | <0.0001 | <0.0001 |

Values are n (%). * Pearson’s χ^2^ test. CON = control group; INT = intervention group.

**ESM Table 3** Independent predictors of baseline to end-of-study changes in WHO-5 scores and WHO-5 score <28 (likely depression).

|  | **WHO-5 score** | | | | **WHO-5 score <28 (likely depression)** | | | |
| --- | --- | --- | --- | --- | --- | --- | --- | --- |
|  | **Beta (SE)** | ***P*** | **Beta (SE)** | ***P*** | **OR (95% CI)** | ***P*** | **OR (95% CI)** | ***P*** |
| **Model 1** |  |  |  |  |  |  |  |  |
| **Age (>65 vs <65 years)** | -0.41 (1.99) | 0.836 | -0.57 (1.84) | 0.757 | 1.38 (0.61;3.13) | 0.444 | 1.27 (0.50;3.20) | 0.614 |
| **Male gender** | 0.59 (2.25) | 0.793 | 0.67 (1.99) | 0.738 | 1.11 (0.46;2.67) | 0.813 | 0.95 (0.35;2.54) | 0.913 |
| **Baseline WHO-5** | 0.64 (0.05) | <0.0001 | 0.63 (0.05) | <.0001 | 0.93 (0.91;0.95) | <0.0001 | 0.93 (0.91;0.96) | <0.0001 |
| **Baseline BMI** | -0.02 (0.18) | 0.934 | -0.01 (0.18) | 0.936 | 1.01 (0.94;1.09) | 0.698 | 1.01 (0.93;1.10) | 0.786 |
| **Baseline HbA_1c_** | 0.81 (0.63) | 0.199 | 0.95 (0.62) | 0.128 | 0.99 (0.77;1.28) | 0.928 | 0.93 (0.68;1.26) | 0.626 |
| **Baseline systolic BP (by 5 mmHg)** | -0.14 (0.22) | 0.539 | -0.28 (0.22) | 0.197 | 1.00 (0.91;1.11) | 0.934 | 1.03 (0.92;1.15) | 0.660 |
| **Baseline VO_2max_** | 0.51 (0.17) | 0.003 |  |  | 0.93 (0.86;0.99) | 0.035 |  |  |
| **Baseline lower body strength (by 10 Nm)** | 0.07 (0.18) | 0.697 |  |  | 0.99 (0.91;1.07) | 0.742 |  |  |
| **Baseline PA volume** |  |  | 1.56 (0.33) | <0.0001 |  |  | 0.79 (0.66;0.95) | 0.011 |
| **Baseline SED-time** |  |  | 3.55 (1.27) | 0.006 |  |  | 0.72 (0.40;1.29) | 0.271 |
| **Model 2** |  |  |  |  |  |  |  |  |
| **Age (>65 vs <65 years)** | -2.94 (1.78) | 0.100 | -2.94 (1.83) | 0.109 | 2.02 (0.93;4.38) | 0.077 | 2.18 (1.05;4.54) | 0.0384 |
| **Male gender** | -2.51 (1.93) | 0.195 | -1.83 (1.99) | 0.359 | 1.59 (0.73;3.46) | 0.248 | 1.56 (0.73;3.31) | 0.252 |
| **Baseline WHO-5** | 0.64 (0.04) | <0.0001 | 0.67 (0.05) | <0.0001 | 0.93 (0.90;0.95) | <0.0001 | 0.93 (0.91;0.95) | <0.0001 |
| **BMI change** | -0.28 (0.49) | 0.568 | -0.24 (0.50) | 0.628 | 1.07 (0.87;1.32) | 0.520 | 1.07 (0.87;1.30) | 0.536 |
| **HbA_1c_ change** | -1.09 (0.68) | 0.108 | -1.24 (0.71) | 0.082 | 1.11 (0.83;1.48) | 0.492 | 1.26 (0.96;1.66) | 0.098 |
| **Systolic BP change** | 0.06 (0.04) | 0.162 | 0.05 (0.04) | 0.258 | 0.99 (0.97;1.00) | 0.114 | 0.99 (0.97;1.01) | 0.245 |
| **VO_2max_ change** | 0.57 (0.23) | 0.014 |  |  | 0.96 (0.87;1.07) | 0.516 |  |  |
| **Lower body strength change** | 0.08 (0.03) | 0.002 |  |  | 0.98 (0.97;0.99) | 0.002 |  |  |
| **PA volume change** |  |  | 1.47 (0.76) | 0.050 | 0.97 (0.71;1.34) | 0.873 | 0.97 (0.71;1.34) | 0.873 |
| **SED-time change** |  |  | 1.67 (2.13) | 0.433 | 0.91 (0.40;2.10) | 0.828 | 0.91 (0.40;2.10) | 0.828 |

WHO = World Health Organization; SE= standard error; BMI = body mass index; HbA_1c_ = glycated haemoglobin; BP = blood pressure; VO_2max_ = maximal oxygen uptake; PA = physical activity; SED-time = sedentary time. Positive and negative beta coefficients indicate positive and negative independent associations, respectively, with baseline to end-of-study changes in WHO-5 scores and WHO-5 score <28.

**ESM Table 4** Independent predictors of baseline to end-of-study changes in PCS and MCS scores.

|  | **PCS score** | | | | **MCS score** | | | |
| --- | --- | --- | --- | --- | --- | --- | --- | --- |
|  | **Beta (SE)** | ***P*** | **Beta (SE)** | ***P*** | **Beta (SE)** | ***P*** | **Beta (SE)** | ***P*** |
| **Model 1** |  |  |  |  |  |  |  |  |
| **Age (>65 vs <65 years)** | 2.30 (1.22) | 0.061 | 2.26 (1.16) | 0.053 | -0.25 (0.94) | 0.787 | 0.37 (0.9) | 0.679 |
| **Male gender** | 0.22 (1.35) | 0.871 | 0.40 (1.16) | 0.732 | 0.03 (1.05) | 0.976 | 1.06 (0.91) | 0.245 |
| **Baseline PCS** | 0.76 (0.06) | <0.0001 | 0.76 (0.06) | <0.0001 | 0.78 (0.05) | <0.0001 | 0.79 (0.05) | <0.0001 |
| **Baseline BMI** | 0.29 (0.11) | 0.009 | 0.31 (0.11) | 0.005 | -0.02 (0.08) | 0.793 | -0.01 (0.08) | 0.937 |
| **Baseline HbA_1c_** | -0.22 (0.37) | 0.560 | -0.07 (0.39) | 0.864 | -0.35 (0.29) | 0.232 | -0.33 (0.30) | 0.282 |
| **Baseline systolic BP (by 5 mmHg)** | 0.14 (0.14) | 0.312 | 0.15 (0.14) | 0.280 | 0.04 (0.11) | 0.712 | 0.06 (0.11) | 0.559 |
| **Baseline VO_2max_** | 0.03 (0.10) | 0.785 |  |  | -0.09 (0.08) | 0.263 |  |  |
| **Baseline lower body strength (by 10 Nm)** | -0.01 (0.11) | 0.909 |  |  | -0.11 (0.09) | 0.227 |  |  |
| **Baseline PA volume** |  |  | -0.02 (0.21) | 0.913 |  |  | 0.03 (0.16) | 0.840 |
| **Baseline SED-time** |  |  | -0.70 (0.79) | 0.379 |  |  | 0.30 (0.62) | 0.631 |
| **Model 2** |  |  |  |  |  |  |  |  |
| **Age (>65 vs <65 years)** | 2.42 (1.24) | 0.053 | 2.26 (1.16) | 0.060 | 0.45 (0.94) | 0.636 | 0.64 (0.94) | 0.496 |
| **Male gender** | 2.45 (1.26) | 0.053 | 0.40 (1.16) | 0.060 | 1.32 (0.95) | 0.169 | 1.29 (0.95) | 0.179 |
| **Baseline PCS** | 0.74 (0.07) | <0.0001 | 0.76 (0.06) | <0.0001 | 0.80 (0.05) | <0.0001 | 0.79 (0.05) | <0.0001 |
| **BMI change** | -0.10 (0.35) | 0.768 | 0.31 (0.11) | 0.818 | 0.15 (0.27) | 0.579 | 0.13 (0.27) | 0.617 |
| **HbA_1c_ change** | 0.84 (0.47) | 0.074 | -0.07 (0.39) | 0.119 | 0.87 (0.35) | 0.014 | 0.91 (0.36) | 0.013 |
| **Systolic BP change** | 0.02 (0.03) | 0.507 | 0.15 (0.14) | 0.562 | -0.02 (0.02) | 0.501 | -0.01 (0.02) | 0.593 |
| **VO_2max_ change** | 0.09 (0.17) | 0.574 |  |  | 0.07 (0.13) | 0.560 |  |  |
| **Lower body strength change** | 0.00 (0.02) | 0.863 |  |  | 0.00 (0.01) | 0.903 |  |  |
| **PA volume change** |  |  | -0.02 (0.21) | 0.933 |  |  | 0.23 (0.38) | 0.548 |
| **SED-time change** |  |  | -0.70 (0.79) | 0.909 |  |  | 0.56 (1.07) | 0.602 |

PCS = physical component summary; MCS = mental component summary; SE= standard error; BMI = body mass index; HbA_1c_ = glycated haemoglobin; BP = blood pressure; VO_2max_ = maximal oxygen uptake; PA = physical activity; SED-time = sedentary time. Positive and negative beta coefficients indicate positive and negative independent associations, respectively, with baseline to end-of-study changes in PCS and MCS scores.

**
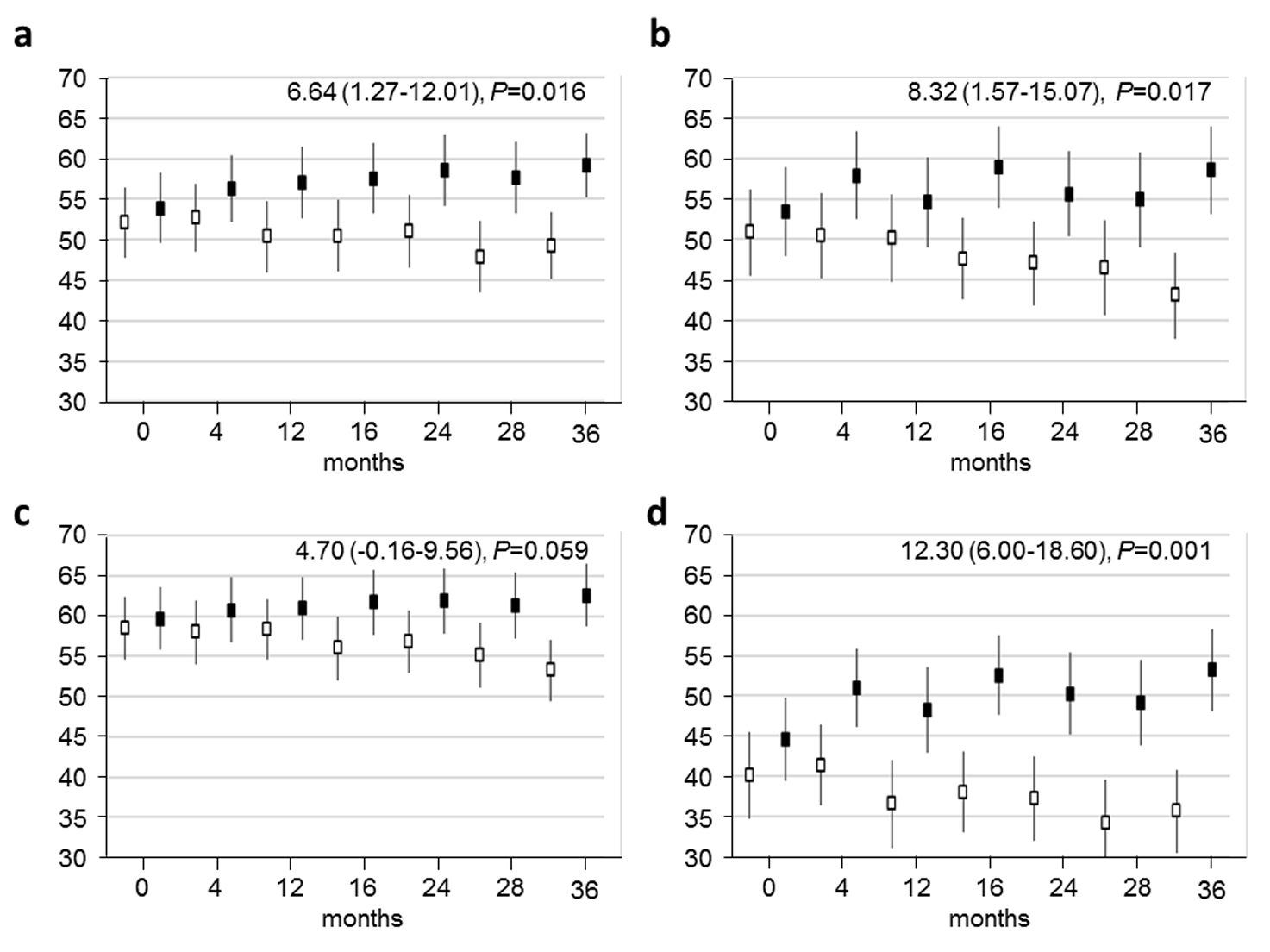
**

**ESM Fig 1 WHO-5 scores over time by age and gender.** Change over time in WHO-5 scores in participants aged < (**a**) and > (**b**) 65 years and in male (**c**) and female (**d**) participants in the INT (black boxes) versus CON (white boxes) group. Data (estimated mean with 95% CI) were calculated on the basis of questionnaires filled in at scheduled visits. The analyses are based on a mixed model for repeated measurements, taking into account within-participant correlation. *P* values were calculated with a mixed model for repeated measurements. Estimated mean differences over time with 95% CIs and *P* values between INT and CON are reported at the top right. WHO = World Health Organization; CON = control group; INT = intervention group.

**
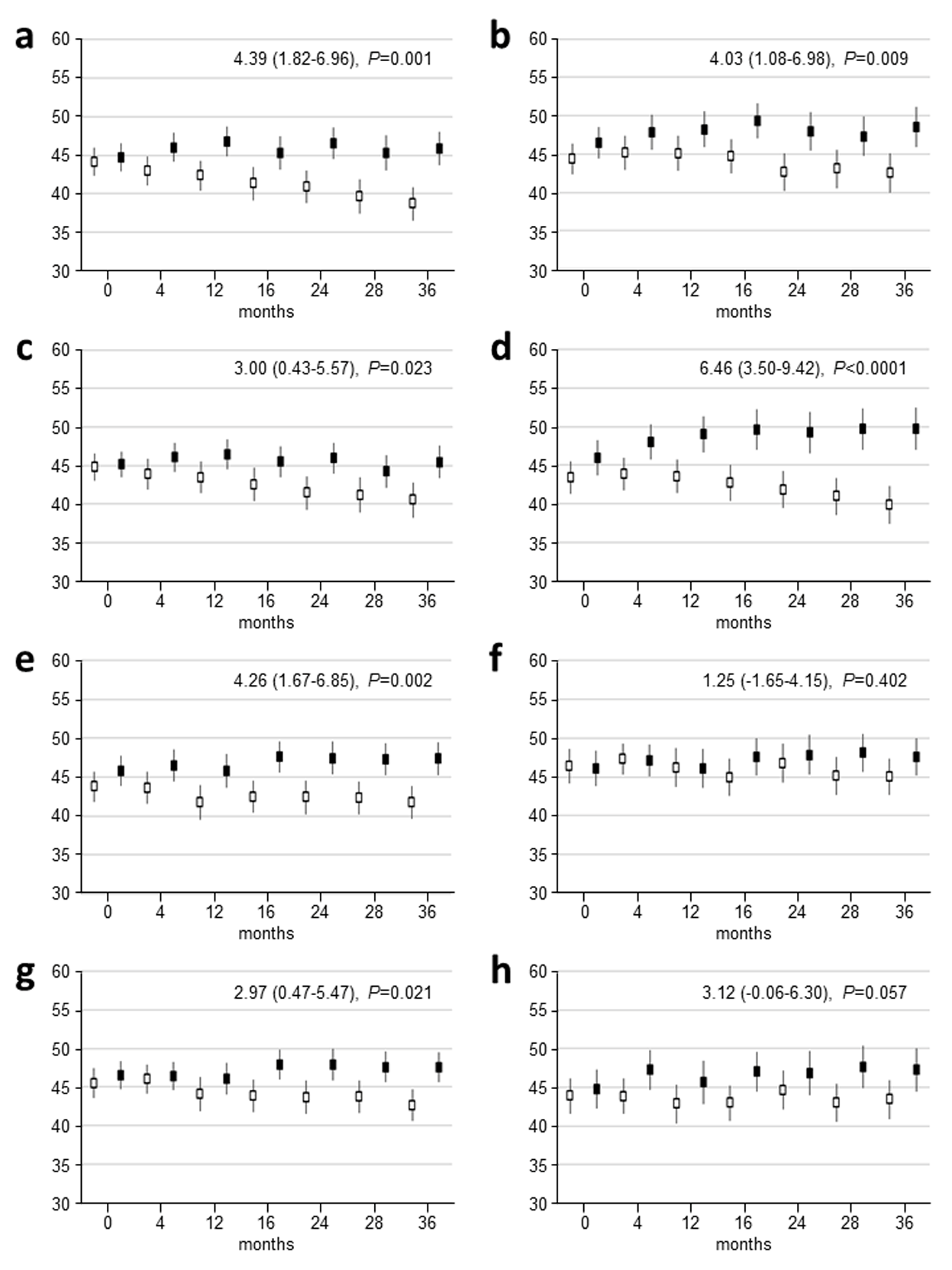
**

**ESM Fig 2 PCS and MCS scores over time by age and gender.** Change over time in PCS and MCS scores in participants aged < (**a**, **e**) and > (**b**, **f**) 65 years and in male (**c**, **g**) and female (**d**, **h**) participants in the INT (black boxes) versus CON (white boxes) group. Data (estimated mean with 95% CI) were calculated on the basis of questionnaires filled in at scheduled visits. The analyses are based on a mixed model for repeated measurements, taking into account within-participant correlation. *P* values were calculated with a mixed model for repeated measurements. Estimated mean differences over time with 95% CIs and *P* values between INT and CON are reported at the top right. PCS = physical component summary; MCS = mental component summary; CON = control group; INT = intervention group.
